# Supplementary material for: MetaRibo-Seq measures translation in microbiomes
Source: Nat Commun. 2020 Jun 29;11:3268. doi: 10.1038/s41467-020-17081-z (PMC7324362; doi:10.1038/s41467-020-17081-z)
Supplement: Supplementary file 10 — Supplementary Data 7 [file 41467_2020_17081_MOESM10_ESM.zip › File2/Confidence_High_Taxonomy/202260_out.krona.html]

Javascript must be enabled to view this page.

members
magnitude
magnitudeUnassigned
count
unassigned
taxon
rank

202260\_out

28

superkingdom
2
28

phylum
976
28

28
class
200643

order
171549
28

family
815
28

28

SRS050941\_contig\_number\_16898SRS147139\_contig\_number\_9538
genus
816
2

1

SRS076976\_contig\_number\_4274
46506
species

93975
species
3

SRS015579\_contig\_number\_16172SRS058723\_contig\_number\_7831SRS893383\_contig\_number\_29143

species
820

SRS016897\_contig\_number\_96SRS018836\_contig\_number\_12624SRS019030\_contig\_number\_10723SRS019286\_contig\_number\_837SRS019445\_contig\_number\_4967SRS019601\_contig\_number\_25228SRS052027\_contig\_number\_13588SRS052078\_contig\_number\_1869SRS077335\_contig\_number\_2012SRS101268\_contig\_number\_19SRS1041033\_contig\_number\_26816SRS1041039\_contig\_number\_23676SRS1041133\_contig\_number\_9762SRS104975\_contig\_number\_1622SRS143895\_contig\_number\_45772SRS147346\_contig\_number\_854SRS147919\_contig\_number\_13075SRS893300\_contig\_number\_3212
18

2

SRS014313\_contig\_number\_2426SRS014683\_contig\_number\_5448
species
47678


SRS148196\_contig\_number\_3791
1
species
2292934


SRS104636\_contig\_number\_17834
1
818
species
